# Locating and applying sociological theories of risk-taking to develop public health interventions for adolescents

**DOI:** 10.1080/14461242.2015.1008537

**Published:** 2015-04-01

**Authors:** Pandora Pound, Rona Campbell

**Affiliations:** ^a^ School of Social and Community Medicine, University of Bristol, Canynge Hall, 39 Whatley Road, BristolBS8 2PS, UK

**Keywords:** sociology, theory, public health, adolescence, interventions, risk-taking

## Abstract

Sociological theories seldom inform public health interventions at the community level. The reasons for this are unclear but may include difficulties in finding, understanding or operationalising theories. We conducted a study to explore the feasibility of locating sociological theories within a specific field of public health, adolescent risk-taking, and to consider their potential for practical application. We identified a range of sociological theories. These explained risk-taking: (i) as being due to lack of social integration; (ii) as a consequence of isolation from mainstream society; (iii) as a rite of passage; (iv) as a response to social constraints; (v) as resistance; (vi) as an aspect of adolescent development; (vii) by the theory of the ‘habitus’; (viii) by situated rationality and social action theories; and (ix) as social practice. We consider these theories in terms of their potential to inform public health interventions for young people.

## Introduction

Adolescence is considered the most significant period for initiating health-related practices that are associated with the largest health burdens in later life (Viner, 2013). There is evidence that activities such as substance use, sexual risk-taking, low levels of physical activity and poor diet co-occur and increase during the teenage years (Spring, Moller, & Coons, 2012) and are associated with poor educational attainment, future morbidity and premature mortality (Kipping, Campbell, MacArthur, Gunnell, & Hickman, 2012). New forms of social media and information technology present additional challenges, with their potential to lead to decreased physical activity, increased exposure to ‘sexting’, cyberbullying (Sawyer et al., 2012), violent and sexualised content, or advertising for cigarettes and alcohol (Viner et al., 2012).

At the same time, key transitions are becoming more protracted and less defined than they were historically, which may result in a lengthened period of vulnerability as adult roles are taken on at a much later age (Catalano et al., 2012; Jackson, Henderson, Frank, & Haw, 2012; Sawyer et al., 2012).

Within public health there is recognition of the importance of health inequalities and other structural, environmental and cultural determinants of adolescent risk-taking. In the case of diet, for example, there is recognition that some environments are ‘obesogenic’ (Lincoln, 2007), while in the context of injury prevention it is acknowledged that strategies to improve the environment are more effective at reducing injury than those attempting to modify young people's behaviour (Michaud, Chandra-Mouli, & Patton, 2009). Furthermore it is recognised that many health-related practices, for example, young peoples' use of substances, or sexual initiation, are influenced by cultural, economic and legal contexts (Michaud et al., 2009; Viner et al., 2012). With regard to social class, the substantial evidence that young people in lower socio-economic positions are more likely to engage in unhealthy practices (Viner et al., 2012) and to continue these into adulthood (Blair, Stewart-Brown, Waterston, & Crowther, 2003; Jackson et al., 2012) is acknowledged, while it is accepted that the strongest determinants of adolescent health are structural factors such as national wealth, income inequality and access to education (Viner et al., 2012).

In terms of reducing adolescent risk-taking, it is acknowledged that relevant public health challenges include measures to end child poverty (Lincoln, 2007), improve living conditions, education and employment opportunities for young women and reduce barriers to youth employment (Viner et al., 2012). In terms of smoking, regulatory interventions such as increasing the price of tobacco, marketing restrictions and mass media interventions are known to have an impact on smoking prevention and cessation among youths (Jackson et al., 2012). Effective controls on the tobacco industry then, but also the alcohol and food industries, are seen as key public health challenges (Lincoln, 2007), since it is recognised that the marketing of unhealthy products and lifestyles targets young people (Sawyer et al., 2012).

Given the widespread acknowledgement of the social factors influencing adolescent risk-taking then, it is perhaps surprising that many community-level public health interventions are still aimed at changing behaviour, an approach that has had only very limited success (Baum & Fisher, 2014; Cohn, 2014). Such interventions tend to draw upon psychological or social-psychological theories (Bonell, Jamal et al., 2013; Bonell, Fletcher et al., 2013; Glanz & Bishop, 2010; NICE 2007) and these generally explain behaviour as individually driven and cognitively motivated (Horrocks & Johnson, 2014). For example, the Gatehouse project, a complex, social intervention within a ‘Health Promoting Schools’ framework, was informed by attachment theory (Bond et al., 2004), while the theory most commonly used to inform Health Promoting School interventions is social cognitive theory (Langford et al., 2014). Baum and Fisher (2014) note that policy is often subject to ‘lifestyle drift’ in that it may start off acknowledging the need to act on the social determinants of health only to eventually drift downstream to focus on individual lifestyle factors. Baum and Fisher suggest this might be because behavioural approaches appeal to governments with neoliberal and individualistic ideologies. Another factor might be that psychological theories, which support the behavioural approach (Horrocks & Johnson, 2014), are more accessible or appear easier to operationalise than sociological theories, despite the latter being undoubtedly more relevant to an upstream approach (Zimmerman, 2013).

Possible reasons why sociological theories are seldom used to inform public health interventions might include difficulty in locating, understanding or applying them. Searching for theory using electronic methods is problematic since much is published in books, which are rarely well indexed on computer databases. Kelly et al. (2010) found that sociological theories and models were missed in electronic searches, particularly if they were more than 25 years old. Sociological theory might also be embedded within empirical work, making it less easy to identify. Some theories are written in an unnecessarily dense and convoluted manner, making them difficult to comprehend. There is also a view that they are not easy to operationalise; for example, Rogers (1968) charged the sociology of medicine with producing complex explanations incapable of being reduced into precise, isolable elements. This perhaps points to the need for more ‘middle-range’ (Merton, 2007), as opposed to ‘grand’, sociological theories, or again, it might simply mean that sociologists need to present and explain their theories more clearly.

We report here on a study that aims to explore the feasibility of locating sociological theories within a specific field of interest, in this case adolescent risk-taking. We begin by describing our efforts to locate the theories, we then present the theories and conclude by considering their relevance for improving the health of young people. Our aim then, is not to conduct a comprehensive search of all relevant sociological theories, but simply to test the ease with which sociological theories can be accessed within a particular field and to consider their potential for practical application.

## Locating the theories

### Search strategy

We felt that the terminology of ‘risk behaviour’ could push us more towards the psychological than the sociological literature so we conceptualised the issue as ‘risk-taking’ (acknowledging that this focus steered us away from wider health-related activity). Because electronic searches are considered unsuitable for locating theoretical literature (Kelly et al., 2010) and because we anticipated that the theories might be framed in a variety of ways and might use unexpected terminology we conducted a manual search. Previous experience of searching for elusive literature suggested we would have missed half the number of relevant papers if we had relied on electronic searches alone (Pound et al., 2005). Since this was an exploratory study we confined our search to two medical sociology journals, *Sociology of Health and Illness* and *Social Science and Medicine*, reasoning that we would be more likely to find sociological theories in these journals than in generic journals of risk. By searching within only two journals we undoubtedly missed some relevant publications; however, our aim was not to conduct an exhaustive search for all relevant theories but to explore the *feasibility* of locating theories.

### Inclusion criteria

We wanted to find sociological theories of risk-taking that either focused on adolescent risk-taking, or had relevance for adolescent risk-taking. Consequently, sociological theories of risk and uncertainty as a feature of postmodernity (e.g., Giddens, 1990, 1999), risk as a product of technological and scientific advancement (Beck, 1992) and sociocultural theories of the concept of risk (Douglas, 1992; Lupton, 1999a; Lupton, 1999b) were *not* the focus of our investigations. We were specifically interested in sociological theories of risk-*taking*, as opposed to theories of risk *per se.* We were aware of sociological reviews of risk-taking (e.g., France, 2000) but these were not of concern to us except insofar as they might alert us to relevant theories. We were also aware of research into lay experiences and perceptions of risk-taking (e.g., Lupton & Tulloch, 2002), but again, these were not relevant to the purpose of our study. We wanted to find sociological *theories* of risk-taking. We therefore excluded generic theories of risk (as opposed to risk-*taking*), review papers and studies that were purely empirical or descriptive. Empirical papers that contained relevant theory were included. We did not use a formal definition of theory, but followed Sutton and Staw (1995) in simply proposing that theory should be about the answer to the question why, and about the connections among phenomena.

### Search process and results

**Figure 1.**
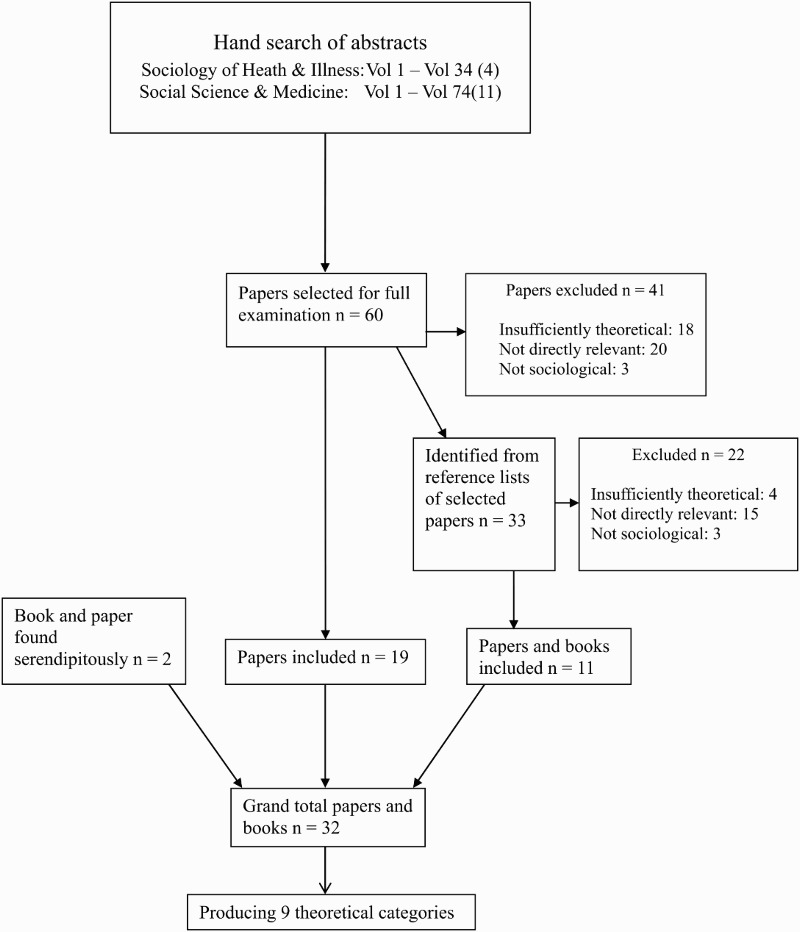
Results of search to locate theories of risk-taking

We hand-searched all the abstracts of all the volumes of the journals *Sociology of Health and Illness* (Volume 1, 1979 to Vol 34(4)) and *Social Science and Medicine* (Volume 1, 1982 to Vol 74(11)) . In cases where the abstracts were insufficiently detailed to determine relevance, the full paper was read. Using our selection criteria 60 papers were identified for full examination, of which 19 were considered relevant (see Figure 1). Promising references from the (60) papers were pursued, a process which produced a further 11 publications. An additional two publications were found serendipitously. In total we identified 32 relevant theoretical papers and books (see Appendix).

Few of the sociological theories were clearly identifiable as such, with only nine of the selected papers containing the word ‘theory’ (or variations of it) in the title. Some papers that appeared at first to be theoretical turned out to be commentaries or critiques. The situation would be improved by sociologists signposting their theories more clearly and also by articulating them more explicitly. However, although it was a lengthy process. a purposive hand-search was appropriate since there turned out to be variation in the terminology employed to conceptualise risk-taking (e.g., deviance, edgework, delinquency) and it would have been difficult to devise a sensitive electronic search without prior knowledge of this terminology. As a check we conducted a retrospective electronic search within the journals *Sociology of Health and Illness* and *Social Science and Medicine*. We found that almost a third (six) of the 19 papers found by hand-searching these journals would have been missed using electronic means only.

## The theories

We grouped the theories into nine broad categories according to similarity of perspective. For example, we grouped theories that linked risk-taking with isolation from mainstream society, even though each might conceptualise this link in a different way. The categories are not mutually exclusive as some overlap with others with regard to a particular aspect of the theory. Some authors enter more than one category, for example, Becker's (1963) theory of deviance relates to the category of theories that are concerned with isolation from mainstream society, but also to the category ‘collective action and social practice’. The theories explained risk-taking: (i) as being due to lack of social integration; (ii) as a consequence of isolation from mainstream society; (iii) as a rite of passage; (iv) as a response to social constraints; (v) as resistance; (vi) as an aspect of adolescent development; (vii) by the theory of the ‘habitus’; (viii) by situated rationality and social action theories; and (ix) as social practice. We present the theories now (summarised in Table 1), before addressing their potential for improving the health of young people.

**Table 1.** Categories of theory relating to risk-taking, identified from the 32 publications.

**1. Lack of social integration**
Durkheim (1952) theorised that strong social ties could protect against an extreme form of risk-taking (suicide), a theory applied to youth suicide (Eckersley & Dear, 2002; Willis, Coombs, Cockerham, & Frison, 2002).
**2. Isolation from mainstream society**
Serious and/or persistent risk-taking is associated with isolation from mainstream society (Douglas & Calvez, 1990; Lightfoot, 1997). Becker (1963) suggests this relationship is causal, i.e., labelling a person as ‘deviant’ results in their isolation, leading them to join a ‘deviant subculture’ and continue risk-taking.
**3. Rites of passage**
In the absence of formal rites of passage (Van Gennep, [1909] 1960) young people in contemporary Western society create their own spontaneous rites of passage which may be harmful (Robb, 1986; Garrett, 1996).
**4. A response to social constraints**
Social and institutional constraints may result in risk-taking (Lyng, 1990, Lyng, 2005) particularly in the context of low social status (Miller, 2005). Risk-taking may be a means of taking control (Denscombe, 2001).
**5. Resistance**
Non-dominant minority groups engage in risk-taking activities as a subtle form of resistance to the dominant group (Factor, Kawachi, & Williams, 2011). Discrimination and alienation increase the likelihood of risk-taking activities, particularly amongst the young. Other, less clearly articulated theories of resistance are provided by Burr (1984), Wearing, Wearing, and Kelly (1994) and Peretti-Watel and Moatti (2006).
**6. An aspect of adolescent development**
Risk-taking is part of the repertoire young people draw upon to construct their identities (Denscombe, 2001; Lightfoot, 1997). Risk-taking (Christensen & Mikkelson, 2008) and narratives about risk-taking (Green, 1997; Lightfoot, 1997) consolidate group membership and mediate group boundaries.
**7. Habitus**
According to Bourdieu (1977, 1984) what people do, i.e., their *practice*, which includes health-related behaviour, is routine but formed in the context of their social location, thereby instilling a view of the world based upon and consistent with their position in it. The *habitus* leads them to perpetuate existing social structures while maintaining a degree of personal agency. Williams (1995), Lindbladh et al. (1996), Lindbladh and Lyttkens (2002), Crawshaw (2004), Dixon and Banwell (2009) apply the theory of the habitus to health-related behaviour and risk-taking. Habituation and routine are as important as calculative thinking in the context of risk-taking (Bloor, 1995).
**8. Situated rationality and social action theories**
A person's risk-taking can be seen as rational when set in the context of the other risks they face. Risk-taking occurs in context of social relationships which in turn are embedded in wider patterns of power and inequality (Rhodes, 1997).
**9. Collective action and social practice**
Risk-taking is not just about an individual's behaviour (Becker, 1963; Chan, Deave, & Greenhalgh, 2010; Delormier, Frohlich, and Potvin, 2009; Frohlich, Corin, & Potvin, 2001, Frohlich, Potvin, Chabot, & Corin, 2002). All aspects of risk-taking should be the focus of research, including those who define activities as ‘risky’, the social institutions that make risk-taking possible, where and how risk-taking occurs, and how it is viewed.

### Lack of social integration

Two papers drew upon Durkheim's theory of social integration to help explain adolescent suicide rates. Durkheim's theory is that the suicide rate varies inversely with the degree of integration of social groups. Thus the social bonds that bind a person to society can protect against suicide. In the case of egoistic suicide, which results from excessive individualism, ‘ … the bond attaching man to life relaxes because that attaching him to society is itself slack’ (1952, pp. 214–215). Willis et al. (2002) focus on Durkheim's proposition that suicide levels increase during periods of rapid societal change because individuals become detached from family and community ties and lose the protective effects of institutions. Willis et al. surmise that adolescents are especially vulnerable since they are in a process of transition and lack the power to manage their environment. Eckersley and Dear (2002) argue that the contemporary Western culture of individualism, which places great value on personal autonomy, independence and self-actualisation, is damaging a growing proportion of young people and may explain the rise in suicide among young men, who are especially vulnerable to the hazards of individualism.

### Isolation from mainstream society

Becker (1963) developed his theory of deviance following his study of marijuana users in the 1960s. Essentially it is a theory of how risk-taking (deviance) may become a permanent way of life. Becker suggests that a crucial step in establishing an enduring pattern of risk-taking is the experience of being labelled as deviant, because a person is then cut off from participation in more conventional groups and may drift into marginal occupations or unemployment. Becker argues that unless a person is able to return to the conventional community early on they will continue down a path of ever-increasing deviance, ending with membership of an organised deviant group. Members of such groups feel a sense of common fate, Becker contends, since they all have to face similar problems. Thus a deviant subculture grows, with a set of world views and ideologies for neutralising conventional norms. The person learns how to continue the deviant behaviour with ease because all the problems of avoiding trouble have already been worked out and there is a stock of lore on relevant subjects which the new member learns.

Lightfoot (1997) distinguishes normative, exploratory, or transitional risk-taking (e.g., experimenting with alcohol) from activities that are health-compromising, destructive or pathogenic (e.g., crack cocaine addiction). She terms the latter ‘marginal risk behaviours’ and her theory is that those engaged in marginal, (i.e., serious or enduring) risk-taking activities also belong to marginal and relatively isolated social groups. Lightfoot argues that groups involved in marginal risk behaviours will have a high degree of internal cohesion and a low degree of permeability to the larger social network, while groups involved in normative risk-taking may also be internally cohesive but will have greater permeability to wider social networks. Both Becker and Lightfoot then, theorise that enduring or serious risk-taking is more likely when a person is isolated from conventional society.

Douglas and Calvez's (1990) theory is that the ongoing dialogue about how to achieve the ideal community engages four different kinds of culture, each of which has a different attitude to the self, risk-taking and the knowledge professions. The ‘centre community’ is hierarchical, holds strong views on correct norms of behaviour and accepts the authority of the established professions. It is very risk-averse and when faced with a threat will aim to exclude all outsiders and repress all deviants. The ‘dissenting enclaves’ espouse equality and protest against the central community. They reject the authority of the central community, suspect professionals and may deride the culture of safety. The ‘individualists’, who are entrepreneurial professionals, are highly idiosyncratic regarding health and diet but are generally risk-takers. Finally the ‘isolates’ tend to be eccentric, fatalistic and expect conspiracy. They reject interference and are idiosyncratic or fatalistic in their attitude to risk. Many are explicit risk-takers in that they may be drug users and/or prostitutes. The dissenting enclaves have a relationship with the centre community, even if it is one of opposition, whereas the individualists have an alliance with the centre community, sharing power and influence. Only the isolates are expelled to the margins by the centre community. Again, as with members of Becker's deviant subcultures and Lightfoot's marginal groups, these people tend to be persistent or serious risk-takers.

### Risk-taking as rite of passage

Two sociologists applied Van Gennep's ([1909] 1960) anthropological theory of rites of passage to adolescent risk-taking. Van Gennep studied the ceremonies that accompany transitions such as death, marriage and coming of age, and classified them into rites of separation, transition and incorporation, hypothesising that they were intended to smooth the potentially disturbing effects of an individual's transition from one social world to another. Robb (1986) drew upon van Gennep's theory to suggest that the uptake of smoking by young people could be understood as an attempted seizing of status in advance, or an ‘anticipatory rite of passage’. Garrett (1996) applied van Gennep's theory to anorexia, suggesting that the illness represents a phase of separation and transition towards a new identity. With recovery the individual is reincorporated into the community and strengthened, she argues, through suffering and confrontation with death. The suggestion is that young people create spontaneous, individual rites of passage, albeit unconsciously, which may be unintentionally harmful.

### Risk-taking as a response to social constraints

Lyng (1990) developed his theory of ‘edgework’ on the basis of a phenomenological study of skydiving. He argued that edgeworkers, in seeking the intense nature and ‘embodied pleasures’ of high-risk experiences, were attempting to transcend, escape or resist the institutional constraints of modern life. In a later work he suggested that, in the context of institutional demands, the practice of exploring limits through voluntary risk-taking could be seen as a personal commitment to freedom and self-creation (Lyng, 2005). Miller (2005) draws upon Lyng's theory of edgework and applies it to adolescents, suggesting that ‘delinquency’ may be a consequence of young people's powerless social status and the constraints to which they are subjected. Miller argues that young people are marginal, disenfranchised and have nearly every aspect of their lives ordered by adults. Many endure punishment, humiliation, physical/sexual abuse or neglect and lack the power to change their situation. Consequently, he suggests, delinquency may be the only opportunity for some adolescents to enjoy authentic, free, creative and self-directed activity. In a similar vein Denscombe (2001) argued that, within a general context of powerlessness, young people who smoke are insisting on their ability to at least control what happens to their body. For young people smoking is not so much about the *appearance* of being in control, he suggests, but about *actually* being in control in the sense of having made a choice, in full knowledge of the risks, ‘ … over something which matters and is recognised to matter by others … ’ (Denscombe, 2001, p. 171).

### Resistance

Factor et al.’s (2011) theory is that non-dominant minority groups (NDMGs) tend to have greater involvement in high-risk behaviours (smoking, alcohol consumption, drug use, poor eating habits, less physical activity, etc.) and that these behaviours represent a form of resistance, whether conscious or unconscious, towards the dominant group. Factor et al. argue that discrimination may result in NDMGs feeling a degree of alienation from the larger society. By engaging in high-risk behaviours NDMGs are able to express their defiance of the dominant group and signal the limits of its power. Everyday acts of resistance may enable non-dominant minorities to express dissatisfaction with their status while avoiding direct negative consequences. Furthermore, argue Factor et al., such groups may develop a collective identity in opposition to the dominant group and may feel pressure not to embrace the attitudes and behaviours of the dominant group, such that if healthy behaviours are associated with the dominant group then engaging with them might incur the hostility of their peers. Factor et al. suggest that NDMG adolescents may engage in unhealthy everyday resistance practices to a greater extent than NDMG adults or adolescents within the majority group.

Earlier and less explicitly formulated theories of risk-taking as resistance include that of Burr (1984) who suggested that the punks and skinhead cultures in the 1980s were symbolic reactions to the dominant culture; Wearing et al. (1994), who hypothesised that smoking amongst young girls could be a means of resisting the submissive ‘good girl’ image; and Peretti-Watel and Moatti (2006) who argued that risk-taking could express resistance to the culture of increased risk awareness and ‘playing it safe’.

### An aspect of adolescent development

Denscombe (2001) argues that in the late modern period a person's identity is less likely to be ascribed by status or institutions meaning that individual choices assume greater significance. He suggests that health-related behaviours form part of the repertoire from which young people draw to construct their identities. Lightfoot (1997) borrows from several disciplines including psychology, sociology, anthropology and folklore to articulate most forcefully the theory that adolescent risk-taking manifests a basic social-psychological process, the development of the social self. Viewed from this perspective, risk-taking can be seen as a declaration of the adolescent's social position in the community and among their peers, while certain patterns of risk-taking are seen to symbolise certain social identities. As Lightfoot (1997, p. 9) explains, ‘Worn like badges – of autonomy, or defiance, or group membership – risks are declarations of the self.’ Furthermore, she observes that teenagers organise risk-taking experiences so that they can be shared. Shared risk-taking, she argues, demonstrates commitment to the group, increases solidarity, promotes trust, is a statement of like-mindedness and a means of initiating new relationships and consolidating existing ones. Christensen and Mikkelsen (2008) likewise argue that engaging with risk is important for children's self-development and provides important emotional and social connections with other children.

Lightfoot also points out that young people tell stories about their risk-taking experiences and that the telling and retelling of these stories can serve to consolidate group membership and mediate group boundaries. In a similar vein Green (1997) theorises that young children use stories about risk and accidents as a resource for constructing social and gendered identities and as a means of delineating the boundaries of peer groups.

### Habitus

Williams (1995) applies Bourdieu's theory of ‘habitus’ to health-related behaviour, valuing its ability to integrate both social structure and personal agency into one theory by focusing on ‘practice’, i.e., what people *do*. According to Bourdieu (1977, 1984), most people do not think about what they do because they do not have to. ‘Practice’, which includes health-related behaviour, is routine, conducted unthinkingly and guided by an implicit logic. The ‘habitus’, explains Williams (1995, pp. 585–6), ‘is formed in the context of people's social locations and inculcates them into a “world view” which is based upon and reconciled to their position, thus serving to reproduce existing social structures. As such, the habitus provides individuals with class-dependent, pre-disposed, yet seemingly “naturalised” ways of thinking, feeling, acting and classifying the social world and their location within it.’ Williams suggests that the theory of the ‘habitus’ can explain how it becomes second nature for people to engage in certain forms of activity and to ‘choose’ different lifestyles, as well as allowing us to understand the relative durability of differing forms of health-related behaviour within social classes. Lindbladh et al. (1996) and Lindlbladh and Lyttkens (2002) have also applied Bourdieu's theory to the field of health-related behaviour, proposing that the habitus shapes health-related decision-making.

Crawshaw (2004) employed the theory of habitus to analyse risk-taking among young working-class men living in an area of high crime, violence and drug use and to explain how these young men's risky practices are continually reproduced. He suggests the existence of a ‘masculine habitus’ which both exposes and predisposes men to risk-taking. Fighting, for example, is sanctioned within the logic of a tough, working-class masculinity and is probably less risky within that context than not fighting, which would be seen as deviating from accepted norms. Dixon and Banwell (2009) draw upon the theory of habitus in their attempt to explain the temporal trends and sub-population variations in smoking behaviour in affluent countries. Noting that high socioeconomic status (SES) males, followed by high SES females, began to abandon smoking once it was adopted by the masses, they suggest the theory of habitus may explain the sedimentation of smoking in successive low SES cohorts.

Relatedly, Bloor (1995) proposes Schutz's theory of ‘systems of relevance’ to interpret variations in risk-taking. Schutz (1970) hypothesised that in a single flash an individual considers various ‘relevances’ when faced with a possible course of action. Schutz does not draw on Bourdieu's theory of habitus, but Bloor suggests that the theory is able to combine habituation and routine behaviour alongside risk calculation to explain an individual's course of action.

### Situated rationality and social action theories

Rhodes (1997) identifies two main theoretical perspectives commonly applied in sociological analyses of sexual risk-taking: situated rationality theories and social action theories. Situated rationality theories posit that risk-taking does not occur in a context-free vacuum but is socially situated, i.e., risks are perceived in the context of other risks and dangers which may be considered more immediate. This highlights the relativity of risk and counters the view of risk-taking as irrational. Social action theories conceptualise risk as the product of social interactions and negotiated actions, shifting the unit of analysis from the individual to the social relationship. In turn social relationships are embedded in wider patterns of power and dominance, so that power differences will influence the activity occurring within them.

### Collective action and social practice

Becker (1963) argued that deviance was located in social processes rather than individual behaviour and that it could not be understood solely by investigating individuals. He suggested instead that we conceptualise deviance as ‘collective action’ and consider all the parties involved. Similarly Frohlich and colleagues develop a theoretical approach over a series of papers (Delormier et al., 2009; Frohlich et al., 2001; Frohlich et al., 2002) that draws upon Bourdieu, among others, to reconceptualise health-related behaviour as ‘social practice’. In a study of smoking initiation they define social practices as the reflexive activities that actors engage in that make and transform the world, the everyday activities that are continually being produced and reproduced (Frohlich et al., 2002). They suggest using the term ‘social practice’ in place of ‘behaviour’ in order to situate the latter in its social context. Analysing smoking initiation in this way requires investigating where people smoke, where they obtain their cigarettes, how smoking is viewed and so on. The social practice approach was adopted by Chan et al. (2010) in an ethnographic study of childhood obesity.

## Implications for reducing adolescent risk-taking

The theories suggest that health-related risk-taking will be more likely among the following young people: those with insufficient ties to their community (Durkheim, 1952; Eckersley & Dear, 2002; Willis et al., 2002); those living in societies without formal rites of passage (Garrett 1996; Robb 1986; Van Gennep 1960); those who are isolated from mainstream society (Becker 1963; Douglas & Calvez 1990; Lightfoot, 1997); those whose lives are overly regulated or circumscribed (Denscombe, 2001; Lyng, 1990, 2005; Miller, 2005); those who suffer discrimination (Factor et al., 2011); those who have low social status (Miller, 2005) and suffer the effects of inequalities (Rhodes, 1997) and; those born into a risk-taking ‘habitus’ (Bourdieu, 1977, 1984; Crawshaw, 2004; Williams, 1995). Exploratory or transitory risk-taking, however, may be a positive aspect of young people's development (Christensen & Mikkelsen, 2008; Denscombe, 2001; Green, 1997; Lightfoot, 1997).

Consequently the theories suggest that the following might protect against adolescent risk-taking: strong social bonds that tie young people to their communities; increased social status, including sufficient freedom to exercise creativity; a reduction in levels of inequality and discrimination; rites of passage (or their contemporary equivalent) to mark the transition to adulthood and; a non-risk-taking ‘habitus’. We now explore in more detail those protective factors that could be addressed at the level of community interventions and consider related evidence.

### Strong social bonds

The theories of Durkheim (1952), Becker (1963), Lightfoot (1997) and Douglas and Calvez (1990) suggest the need for interventions to integrate young people in danger of becoming isolated or of drifting away from mainstream society, rather than punitive measures that would expel them to the margins of conventional society. They lend theoretical support to targeted interventions that support vulnerable young people, as well as universal interventions that aim to tie young people more closely to their communities. These theories relating to the need for social connectedness appear to have empirical support. Resnick et al. (1997) found that family-connectedness and perceived school-connectedness were protective against every measure of health-related risk-taking except a history of pregnancy. Adolescents who feel connected to their family are more likely to delay sexual initiation, report lower levels of substance use and less likely to engage in violence (Viner et al., 2012). A child's sense of belonging and connectedness to their school, a sense of neighbourhood belonging and parental involvement are all related to lower engagement in health-related risk-taking (Brooks, Magnusson, Spencer, & Morgan, 2012). A whole-school intervention that aimed to increase children's sense of attachment and connectedness reduced health-related risk taking by 25% (Patton et al., 2006). Community-level interventions to decrease risk-taking by enhancing social connectedness and tying young people more closely to their communities would thus appear to have good theoretical and empirical support. The concepts of social integration, connectedness and belonging are related to the notion of social capital (Almedon, 2005) that sits within an assets-based approach emphasising the importance of enhancing the protective factors in young people's lives (Viner et al., 2012).

### Increased social status

Miller (2005) suggested that young peoples' powerless social status and the constraints to which they are subject by virtue of their age, mean that ‘adolescent delinquency’ may be the only opportunity some young people have for self-directed activity. Similarly Denscombe (2001) argues that smoking may be one of the few important things that young people, within a general context of powerlessness, feel they can make a choice about. These theories suggest the need to improve the social status of young people, not just in relation to their class, gender and other aspects of social inequality, but in relation to the particular context of their age. Christensen (2004) suggests that a different approach to the study of childhood is needed, one that acknowledges children's involvement in managing their own and others’ health and explores how children actively contribute to their families and communities. Lightfoot's (1997) perspective, in which she conceptualises exploratory risk-taking as a way for adolescents to creatively manage their relationships and identities, can be seen as an example of this more positive, salutogenic approach.

The association between adolescent risk-taking and lower socio-economic class (Blair et al., 2003; Jackson et al., 2012; Viner et al., 2012) is elucidated, at least in part, by Factor et al.’s (2011) theory of resistance, which proposes that non-dominant minority groups' greater involvement in health-related risk taking is a means of resisting, and of ensuring non-alignment with, the dominant social group, a dynamic that applies especially strongly to non-dominant minority adolescents. Similarly, situated rationality theories and social action theories (Rhodes, 1997) help draw out the association between inequalities and adolescent risk-taking, by showing how inequities within social relationships can impel those with less power to take risks (for example, with sexual risk-taking) or limit their ability to make healthy and safe choices. Finally, as Williams (1995) notes, Bourdieu's theory of ‘habitus’ helps explain the durability of class-related forms of risk-taking by illustrating how distinct practices and ways of being are deeply embedded in certain cultures and reproduced almost automatically over generations.

While community-level interventions would not be able to address the root causes of inequality and discrimination that many young people suffer, they could potentially improve the social status of young people by allowing them the opportunity for self-directed and creative activity. Interventions that enable young people to contribute actively to their communities would not only be likely to increase their confidence and sense of achievement, but would help to recast young people as active, caregiving members of their communities, rather than as potentially troublesome dependents.

### Rites of passage

The relationship between the absence of rites of passage and the presence of risk-taking was only suggestive in the literature we located but Le Breton (2004), whom we discovered after following this lead, makes a forceful case in its support. He argues that adolescent risk-taking within contemporary Western societies can be understood as a self-initiated rite of passage, but whereas in traditional societies rites of passage are communal and conducted under the supervision of elders to ensure community affiliation, in contemporary Western societies risk-taking tends to occur privately or among peers and often takes place in the context of ruptures in social relationships rather than in affirmation of them.

If risk-taking can be seen as a self-initiated rite of passage, it might follow that formal rites of passage could protect against risk-taking. Because they acknowledge, celebrate and strengthen a person's bond to their community, rites of passage – or their contemporary equivalent – might reduce the possibility of isolation and alienation. Similarly, by publicly displaying respect for a young person and communally acknowledging their passage to adulthood, rites of passage could positively contribute to the social status of that person. In theory then, they could protect against some of the determinants of risk-taking that our sociological theories identified. However, Larson and Martin (2012) note that traditional rites also contain an element of challenge and here they can be seen to relate to the concept of resilience. Drawing upon the work of Rutter (1985, 1987), Davies (2013) suggests that just as vaccination exposes children to a small amount of pathogen to enable an immune response to be mounted if necessary, so too we need to inoculate young people against adversity by exposing them to low doses of challenge in safe and supported environments, to foster resilience. Resilience has been variously described as the ability to recover from negative events (Olsson, Bond, Burns, Vella-Brodrick, & Sawyer, 2003) or to rebound after experiencing hardship (Wexler, DiFluvio, & Burke, 2009). Could it be that programmes aiming to develop resilience are a contemporary form of traditional rites of passage? If so, we suggest they merit serious consideration. Viner (2013) observes that the naturally occurring transitions in adolescence are ideal points for interventions aiming to build resilience in young people. If traditional rites of passage for young people had the potential to promote resilience, strengthen social bonds, smooth transitions and increase social status, it should not be beyond us to develop a contemporary equivalent that could deliver the same benefits and so protect against risk-taking in young people.

## Conclusion

As we noted in the introduction, it is acknowledged within public health that social factors influence adolescent risk-taking. The sociological theories we identify here, however, provide a theoretical framework for understanding *how* these social factors are influential and the various levels on which they may operate. The theories are wide-ranging and fall into a range of theoretical traditions including cultural theory, functionalism, conflict theory, labelling theory and symbolic interactionism. At one end of the scale they suggest that while society does not ‘cause’ risk-taking, it can nevertheless produce conditions that favour risk-taking, particularly if those conditions result in individuals or social groups becoming isolated and detached from the mainstream (Becker, 1963; Durkheim, 1952). At the other end of the scale the act of smoking is analysed as part of the repertoire that young people draw upon to construct their identities (Denscombe, 2001). The theories remind us that equality, increased social status for adolescents, strong social ties and freedom from over-constraint, are all factors that protect against adolescent risk-taking. At the same time they point to the positive functions of risk-taking, as a means of consolidating group membership, contributing to the development of identity and providing young people with the possibility of free, authentic expression. By consistently embedding risk-taking within its social context and emphasising that risk-taking is not something a single individual does, but is ‘collective action’, or ‘social practice’, the theories both relieve us of the burden of judging young people for their risk-taking and enable a greater collective responsibility for the conditions that give rise to risk-taking.

We conclude from this exploratory study that although time-consuming, it is possible to locate relevant sociological theories within a specific field of public health. The theories we found have the potential to lead to improvements in adolescent health, not only by improving our comprehension of the factors and pathways involved in adolescent risk-taking, but by providing the depth of understanding and insight necessary to design effective community-level interventions that take account of the social determinants of risk-taking. Nevertheless, it was difficult to locate some of the theories, raising the possibility that they could have been more clearly signposted to increase their accessibility to those outside sociology. Unless sociological theories are made more accessible there is a danger that the opportunity for merging theory and practice will be lost. As a result sociology might be ignored by policymakers (Turner, 1991, 1998) and the world outside sociology will be the poorer for it.

## Appendix: publications identified in search (*n* = 32)

Becker, H. (1963). *Outsiders*. New York: The Free Press.

Bloor, M. (1995). A user's guide to contrasting theories of HIV-related risk behaviour. In J. Gabe (Ed.), *Medicine, health and risk: Sociological approaches* (pp. 19–30). Oxford: Blackwell.

Bourdieu, P. (1977). *Outline of a theory of practice*. Cambridge: Cambridge University Press.

Bourdieu, P. (1984). *Distinction: a social critique of the judgement of taste*. London: Routledge.

Burr, A. (1984). The ideologies of despair: a symbolic interpretation of punks and skinheads' usage of barbiturates. *Social Science and Medicine*, *19*(9), 929–938.

Chan, C., Deave, T., & Greenhalgh, T. (2010). Childhood obesity in transition zones: An analysis using structuration theory. *Sociology of Health and Illness*, 32(5), 711–729.

Christensen, P., & Mikkelsen, M. (2008). Jumping off and being careful: Children's strategies of risk management in everyday life. *Sociology of Health and Illness*, *30*(1), 112–130.

Crawshaw, P. (2004). The logic of practice in the risky community: The potential of the work of Pierre Bourdieu for theorising young men's risk-taking.” In W. Mitchell & R. Bunton (Eds.), *Young people, risk and leisure: Constructing identities in everyday life* (pp. 224–242). Houndsmills: Palgrave Macmillan.

Delormier, T, Frohlich, K, Potvin, L. (2009). Food and eating as social practice – Understanding eating patterns as social phenomena and implications for public health. *Sociology of Health and Illness, 31*(*2*), 215–228.

Denscombe, M. (2001). Uncertain identities and health-risking behaviour: The case of young people and smoking in late modernity. *British journal of Sociology*, *52*(1), 157–177.

Dixon, J., & Banwell, C. (2009). Theory driven research designs for explaining behavioural health risk transitions: The case of smoking. *Social Science and Medicine*, *68*, 2206–2214.

Douglas, M., & Marcel, C. (1990). The self as risk-taker: A cultural theory of contagion in relation to AIDS. *Sociological Review*, *38*, 445–464.

Durkheim, E. (First published in England 1952). *Suicide*. (J. Spaulding and G. Simpson Trans.). London: Routledge and Kegan Paul.

Eckersley, R., & Dear, K. (2002). Cultural correlates of youth suicide. *Social Science and Medicine*, *55*, 1891–1904.

Factor, R., Kawachi, I., & Williams, D. (2011). Understanding high-risk behaviour among non-dominant minorities: A social resistance framework. *Social Science and Medicine*, *73*, 1292–1301.

Frohlich, K., Corin, E., & Potvin, L. (2001). A theoretical proposal for the relationship between context and disease. *Sociology of Health and Illness*, *23*(*6*), 776–797.

Frohlich, K., Potvin, L., Chabot, P., & Corin, E. (2002). A theoretical and empirical analysis of context: Neighbourhoods, smoking and youth. *Social Science and Medicine*, *54*, 1401–1417.

Garrett, C. (1996). Recovery from anorexia nervosa: A Durkheimian interpretation. *Social Science and Medicine*, *43*(*10*), 1489–1506.

Green, J. (1997). Risk and the construction of social identity: Children's talk about accidents. *Sociology of Health and Illness*, *19*(*4*), 457–479.

Lindbladh, E., Lyttkens, C., Hanson, B., Ostergren, P., Isacsson, S., & Lindgren, B. (1996). An economic and sociological interpretation of social differences in health-related behaviour: An encounter as a guide to social epidemiology. *Social Science and Medicine*, *43*(*12*), 1817–1827.

Lindbladh, E., & Lyttkens, C. (2002). Habit versus choice: The process of decision-making in health-related behaviour. *Social Science and Medicine*, *55*, 451–465.

Lightfoot, C. (1997). *The culture of adolescent risk-taking.* New York: Guilford Press.

Lyng, S. (1990). Edgework: A social psychological analysis of voluntary risk-taking. *American Journal of Sociology*, *94*(*4*), 851–886.

Lyng, S. (2005). *Edgework. The sociology of risk-taking*. (Ed) New York: Routledge.

Miller, W. (2005). Adolescents on the edge: The sensual side of delinquency. In S. Lyng (Ed.). *Edgework. The Sociology of Risk-taking* (pp. 153–172). New York: Routledge.

Peretti-Watel, P., & Moatti, J. (2006). Understanding risk behaviours: How the sociology of deviance may contribute? The case of drug-taking. *Social Science and Medicine*, *63*, 675–679.

Rhodes, T. (1997). Risk theory in epidemic times: Sex, drugs and the social organisation of ‘risk behaviour’. *Sociology of Health and Illness*, *19*, 208–227.

Robb, J. (1986). Smoking as an anticipatory rite of passage: some sociological hypotheses on health-related behaviour. *Social Science and Medicine*, *23*(*6*), 621–627.

Van Gennep, A. ([1909] 1960). *The Rites of Passage*. London: Routledge.

Wearing, B., Wearing, S., & Kelly, K. (1994). Adolescent women, identity and smoking: Leisure experience as resistance. *Sociology of Health and Illness*, *16*(5), 626–643.

Williams, S. (1995). Theorising class, health and lifestyles: Can Bourdieu help us? *Sociology of Health and Illness*, *17*(5), 577–604.

Willis, L., Coombs, D., Cockerham, W., & Frison, S. (2002). Ready to die: A postmodern interpretation of the increase of African-American adolescent male suicide. *Social Science and Medicine*, *55*, 907–920.

## References

[CIT0001] Almedon A. M. (2005). Social capital and mental health: An interdisciplinary review of primary evidence. *Social Science and Medicine*.

[CIT0002] Baum F., Fisher M. (2014). Why behavioural health promotion endures despite its failure to reduce health inequities. *Sociology of Health and Illness*.

[CIT0003] Beck U. (1992). *Risk society: Towards a new modernity*.

[CIT0004] Becker H. (1963). *Outsiders*.

[CIT0005] Blair M., Stewart-Brown S., Waterston T., Crowther R. (2003). *Child public health.*.

[CIT0006] Bloor M., Gabe J. (1995). A user's guide to contrasting theories of HIV-related risk behaviour. *Medicine, health and risk: Sociological approaches*.

[CIT0007] Bond L., Patton G., Glover S., Carlin J., Butler H., Thomas L., Bowes G. (2004). The Gatehouse Project: Can a multilevel school intervention affect emotional wellbeing and health risk behaviours?. *Journal of Epidemiology and Community Health*.

[CIT0008] Bonell C., Fletcher A., Jamal F., Wells H., Harden A., Murphy S., Thomas J. (2013). Theories of how the school environment impacts on student health: Systematic review and synthesis. *Health and Place*.

[CIT0009] Bonell C., Jamal F., Harden A., Wells H., Parry W., Fletcher A., Petticrew M., Thomas J., Whitehead M., Campbell R., Murphy S., Moore L. (2013). Systematic review of the effects of schools and school environment interventions on health: Evidence mapping and synthesis. *Public Health Research*.

[CIT0010] Bourdieu P. (1977). *Outline of a theory of practice*.

[CIT0011] Bourdieu P. (1984). *Distinction: a social critique of the judgement of taste*.

[CIT0012] Brooks F., Magnusson J., Spencer N., Morgan A. (2012). Adolescent multiple risk behaviour: An asset approach to the role of family, school and community. *Journal of Public Health*.

[CIT0013] Burr A. (1984). The ideologies of despair: A symbolic interpretation of punks and skinheads’ usage of barbiturates. *Social Science and Medicine*.

[CIT0014] Catalano R., Fagan A., Gavin L., Greenberg M., Irwin C., Ross D., Shek D. (2012). Worldwide application of prevention science in adolescent health. *The Lancet*.

[CIT0015] Chan C., Deave T., Greenhalgh T. (2010). Childhood obesity in transition zones: An analysis using structuration theory. *Sociology of Health and Illness*.

[CIT0016] Christensen P. (2004). The health-promoting family: A conceptual framework for future research. *Social Science and Medicine*.

[CIT0017] Christensen P., Mikkelsen M. (2008). Jumping off and being careful: children's strategies of risk management in everyday life. *Sociology of Health and Illness*.

[CIT0018] Cohn S. (2014). From health behaviours to health practices: An introduction. *Sociology of Health and Illness*.

[CIT0019] Crawshaw P., Mitchell W., Bunton R. (2004). The logic of practice in the risky community: The potential of the work of Pierre Bourdieu for theorising young men's risk-taking. *Young people, risk and leisure: Constructing identities in everyday life*.

[CIT0020] Davies S. (2013). *Annual report of the chief medical officer 2012. Our children deserve better: Prevention pays*.

[CIT0023] Delormier T., Frohlich K., Potvin L. (2009). Food and eating as social practice – Understanding eating patterns as social phenomena and implications for public health. *Sociology of Health and Illness*.

[CIT0024] Denscombe M. (2001). Uncertain identities and health-risking behaviour: The case of young people and smoking in late modernity. *British journal of Sociology*.

[CIT0025] Dixon J., Banwell C. (2009). Theory driven research designs for explaining behavioural health risk transitions: The case of smoking. *Social Science and Medicine*.

[CIT0026] Douglas M. (1992). *Risk and blame: Essays in cultural theory*.

[CIT0027] Douglas M., Calvez M. (1990). The self as risk-taker: A cultural theory of contagion in relation to AIDS. *The Sociological Review*.

[CIT0028] Durkheim E. (First published in England 1952). *Suicide*.

[CIT0029] Eckersley R., Dear K. (2002). Cultural correlates of youth suicide. *Social Science and Medicine*.

[CIT0030] Factor R., Kawachi I., Williams D. (2011). Understanding high-risk behaviour among non-dominant minorities: A social resistance framework. *Social Science and Medicine*.

[CIT0031] France A. (2000). Towards a sociological understanding of youth and their risk-taking. *Journal of Youth Studies*.

[CIT0032] Frohlich K., Corin E., Potvin L. (2001). A theoretical proposal for the relationship between context and disease. *Sociology of Health and Illness*.

[CIT0033] Frohlich K., Potvin L., Chabot P., Corin E. (2002). A theoretical and empirical analysis of context: Neighbourhoods, smoking and youth. *Social Science and Medicine*.

[CIT0034] Garrett C. (1996). Recovery from anorexia nervosa: A Durkheimian interpretation. *Social Science and Medicine*.

[CIT0035] Giddens A. (1990). *The consequences of modernity*.

[CIT0036] Giddens A. (1999). Risk and responsibility. *The Modern Law Review*.

[CIT0037] Glanz K., Bishop D. (2010). The role of behavioural science theory in development and implementation of public health interventions. *Annual Review Public Health*.

[CIT0038] Green J. (1997). Risk and the construction of social identity: Children's talk about accidents. *Sociology of Health and Illness*.

[CIT0039] Horrocks C., Johnson S. (2014). A socially situated approach to inform ways to improve health and wellbeing. *Sociology of Health and Illness*.

[CIT0040] Jackson C., Henderson M., Frank J., Haw S. (2012). An overview of prevention of multiple risk behaviour in adolescence and young adulthood. *Journal of Public Health*.

[CIT0041] Kelly M., Morgan A., Ellis S., Younger T., Huntley J., Swann C. (2010). Evidence based public health: A review of the experience of the National Institute of Health and Clinical Excellence (NICE) of developing public health guidance in England. *Social Science and Medicine*.

[CIT0042] Kipping R., Campbell R., MacArthur G., Gunnell D., Hickman M. (2012). Multiple risk behaviours in adolescence. *Journal of Public Health*.

[CIT0043] Langford R., Bonell C. P., Jones H. E., Pouliou T., Murphy S. M., Waters E., Komro K. A., Gibbs L. F., Magnus D., Campbell R. (2014).

[CIT0044] Larson S., Martin L. (2012). Risk taking and rites of passage. *Reclaiming Children and Youth*.

[CIT0045] Le Breton D. (2004). The anthropology of adolescent risk-taking behaviours. *Body and Society*.

[CIT0046] Lightfoot C. (1997). *The culture of adolescent risk-taking.*.

[CIT0047] Lincoln P., Griffiths S., Hunter D. (2007). Healthy childhood and a life-course approach to public health. *New perspectives in public health*.

[CIT0048] Lindbladh E., Lyttkens C. (2002). Habit versus choice: The process of decision-making in health-related behaviour. *Social Science and Medicine*.

[CIT0049] Lindbladh E., Lyttkens C., Hanson B. S., Ostergren P., Isacsson S., Lindgren B. (1996). An economic and sociological interpretation of social differences in health-related behaviour: An encounter as a guide to social epidemiology. *Social Science and Medicine*.

[CIT0050] Lupton D. (1999a). *Risk and sociocultural theory: New directions and perspectives*.

[CIT0051] Lupton D. (1999b). *Risk*.

[CIT0052] Lupton D., Tulloch J. (2002). Life would be pretty dull without risk: Voluntary risk-taking and its pleasures. *Health, Risk & Society*.

[CIT0053] Lyng S. (1990). Edgework: A social psychological analysis of voluntary risk-taking. *American Journal of Sociology*.

[CIT0054] Lyng S. (2005). *Edgework. The sociology of risk-taking*.

[CIT0055] Merton R., Calhoun C., Gerteis J., Moody J., Pfaff S., Virk I. ([1949] 2007). On sociological theories of the middle range. *Classical sociological theory*.

[CIT0056] Michaud P., Chandra-Mouli V., Patton G., Detels R., Beaglehole R., Langsang M. A., Gulliford M. (2009). Adolescent health. *The oxford textbook of public health*.

[CIT0057] Miller W., Lyng S. (2005). Adolescents on the edge: The sensual side of delinquency. *Edgework. The sociology of risk-taking*.

[CIT0058] National Institute of Health and Clinical Effectiveness (NICE). (2007). http://www.nice.org.uk/nicemedia/live/11868/37987/37987.pdf.

[CIT0059] Olsson C., Bond L., Burns J., Vella-Brodrick D., Sawyer S. (2003). Adolescent resilience: A concept analysis. *Journal of Adolescence*.

[CIT0060] Patton G., Bond L., Carlin J., Thomas L., Butler H., Glover S., Catalano R., Bowes G. (2006). Promoting social inclusion in schools: A group-randomized trial of effects of student health risk behaviour and well-being. *American Journal of Public Health*.

[CIT0061] Peretti-Watel P., Moatti J. (2006). Understanding risk behaviours: How the sociology of deviance may contribute? The case of drug-taking. *Social Science and Medicine*.

[CIT0062] Pound P., Britten N., Morgan M., Yardley L., Pope C., Daker-White G., Campbell R. (2005). Resisting medicines: A synthesis of qualitative studies of medicine taking. *Social Science and Medicine*.

[CIT0063] Resnick M., Bearman P., Blum R., Bauman K., Harris K., Jones J., Tabor J., Beuhring T., Sieving R., Shew M., Ireland M., Bearinger L., Udry J. (1997). Protecting adolescents from harm. Findings from the national longitudinal study on adolescent health. *JAMA*.

[CIT0064] Rhodes T. (1997). Risk theory in epidemic times: sex, drugs and the social organisation of ‘risk behaviour’. *Sociology of Health and Illness*.

[CIT0065] Robb J. (1986). Smoking as an anticipatory rite of passage: Some sociological hypotheses on health-related behaviour. *Social Science and Medicine*.

[CIT0066] Rogers E. (1968). Public Health asks of sociology … Can the health sciences resolve society's problems in the absence of a science of human values and goals?. *Science*.

[CIT0067] Rutter M. (1985). Resilience in the face of adversity. *The British Journal of Psychiatry*.

[CIT0068] Rutter M. (1987). Psychosocial resilience and protective mechanisms. *American Journal of Orthopsychiatry*.

[CIT0069] Sawyer S., Afifi R., Bearinger L., Blakemore S., Dick B., Ezeh A., Patton G. (2012). Adolescence: A foundation for future health. *The Lancet*.

[CIT0070] Schutz A. (1970). *Reflections on the problem of relevance*.

[CIT0071] Spring B., Moller A., Coons M. (2012). Multiple health behaviours: Overview and implications. *Journal of Public Health*.

[CIT0072] Sutton R., Staw B. (1995). What theory is not. *Administrative Science Quarterly*.

[CIT0073] Turner J. (1991). Developing cumulative and practical knowledge through metatheorizing. *Sociological Perspectives*.

[CIT0074] Turner J. (1998). Must sociological theory and sociological practice be so far apart? A Polemical Answer. *Sociological Perspectives*.

[CIT0075] Van Gennep A. ([1909] 1960). *The rites of passage*.

[CIT0076] Viner R., Davies S. (2013). Life stage: Adolescence. *Annual report of the chief medical officer 2012. Our children deserve better: Prevention pays* (Chapter 8, pp. 1–11).

[CIT0077] Viner R., Ozer E., Denny S., Marmot M., Resnick M., Fatusi A., Currie C. (2012). Adolescence and the social determinants of health. *The Lancet*.

[CIT0078] Wearing B., Wearing S., Kelly K. (1994). Adolescent women, identity and smoking: Leisure experience as resistance. *Sociology of Health and Illness*.

[CIT0079] Wexler L., DiFluvio G., Burke T. (2009). Resilience and marginalized youth: Making a case for personal and collective meaning-making as part of resilience research in public health. *Social Science and Medicine*.

[CIT0080] Williams S. (1995). Theorising class, health and lifestyles: Can Bourdieu help us?. *Sociology of Health and Illness*.

[CIT0081] Willis L., Coombs D., Cockerham W., Frison S. (2002). Ready to die: A postmodern interpretation of the increase of African-American adolescent male suicide. *Social Science and Medicine*.

[CIT0082] Zimmerman F. (2013). Habit, custom and power: A multi-level theory of population health. *Social Science and Medicine*.

